# Analytic Process and Linguistic Style: Exploring Analysts’ Treatment Notes in the Light of Linguistic Measures of the Referential Process

**DOI:** 10.1007/s10936-021-09771-6

**Published:** 2021-02-02

**Authors:** Rachele Mariani, Leon Hoffman

**Affiliations:** 1 Department of Dynamic and Clinical Psychology, Sapienza University, Rome, Italy; 2 Pacella Research Center, New York Psychoanalytic Society and Institute, New York City, NY USA

**Keywords:** Referential activity, Psychoanalytic process, Linguistic style, Psychoanalyst’s notes, Linguistic measures, Computerized measures

## Abstract

This paper presents a comparison between a clinical evaluation and a computerized linguistic analysis of the treatment notes of the first two years of an analysis conducted four sessions a week with the patient lying on a couch. Clinical notes had been written as part of the analyst’s standard practice after every session, some years prior to the planning of this study. The notes describe the analytic interchange and the analyst’s internal thoughts. The linguistic analysis focuses on two analytically relevant linguistic variables: Referential Activity (RA), a measure of the degree of connection between emotional processing and language, and Reflection, the use of words referring to thoughts. The examination of the linguistic measures point to overlooked parts of sessions which may be clinically significant. In particular, the examination of the clinical material during the nodal points of the first summer break, where significant changes in the linguistic measures were seen, provided clinical understanding of the analytic work that was not explicitly noted at the time of treatment. This method has the potential to be utilized in ongoing treatments and to improve the supervisory process.

## Introduction

The value of the empirical study of psychoanalytic treatment has been widely disputed. Many analysts as well as researchers argue that systematic empirical research, including process as well as outcome research, is necessary for the development of the field. Some of this work is summarized in the Open-Door Review of empirical studies (Leuzinger-Bohleber and Kächele 2015). Others, however, argue that empirical research is at best unnecessary, and at worst has the potential to interfere with analytic work; and particularly that the efficacy of the analytic process may be nullified by recording sessions. (Green 2003).

Tuckett (2000) has noted that psychoanalytic data refer to “*the occurrences noted by the analyst in the session,* provided they are apprehended within the framework of free-floating attention and free association” (p. 406, emphasis in original; also cited in Bucci (2007, p. 620). In contrast to some analysts who emphasize the centrality of the analyst’s subjectivity, Tuckett is not opposed to recordings of sessions; as he says:

It may well be interesting to use them to see what is noted and what is not and to explore what the analyst has to say about that. But the essence of psychoanalysis is that the analyst, as a receptive human being making sense within a communicative field, unconsciously (as well as consciously) picks up the data within a framework of meanings. A subjective report rather than an “objective” transcript is therefore indispensable as the basic data [Tuckett 2000, p. 407].

Marianne Leuzinger-Bohleber (2015) emphasizes the importance of psychoanalytic research, and also differentiates between different types of research. She says:

Today we can differentiate between two different groups of psychoanalytic research, the clinical and the extra-clinical. By clinical research is meant genuine psychoanalytic research in the psychoanalytic situation itself. Ulrich Moser (2009) describes this as on-line research whereas, as outlined below, the extra-clinical research (the off-line research) occurs after the psychoanalytic sessions and embraces a variety of research strategies (p. 21).

Prior work has illustrated the value of comparing *clinical* evaluations of psychotherapeutic treatments with *extra-clinical* or *systematic empirical* evaluations of the same recorded and transcribed psychotherapeutic treatment (for example, Jones and Windholz 1990). Bucci and Maskit (2007) have demonstrated agreement between the psychoanalytic conceptions of recorded clinical work and the independent application of computerized linguistic measures. Recent studies from the Referential Process Research Group have provided new and interesting possibilities for enhancing the evaluation of the therapeutic process through linguistic analysis of psychoanalysts' notes and reports (Bucci et al. 2012; Hoffman et al. 2013).

In this exploratory study, we expand on that work by illustrating the potential clinical value of a systematic examination of computerized linguistic measures applied to ongoing process notes by the treating analyst herself. The central claim of the research discussed here is that it is possible to combine a clinical perspective with a systematic empirical approach by applying linguistic measures to the analyst’s notes. This approach permits an integration of the analyst’s subjective appraisal with objective assessment of the treatment process. We claim that this approach can potentially be of value not only from the perspective of research, but also as an aid to the clinician in conducting the treatment.

As Bucci (2007) has noted, there are four experiential domains in play during the analytic interaction: the conscious perspectives of the analyst and of the patient, and the ongoing experience within the analyst and within the patient of which they are not aware. It is not generally recognized that the case report, which has been the dominant source of clinical knowledge in psychoanalysis, represents only one aspect of these domains, and that only partially: the perspective of the analyst that he is able (and willing) to bring to mind and to articulate in words. The approach to be discussed in this paper provides additional perspectives: the subjective, sometimes unrecognized experience of the analyst as well as some aspects of the interaction between analyst and patient, which may be revealed through linguistic analysis of the analyst's notes. This analysis potentially allows uncovering of aspects of the analytic interchange that may not have been explicitly recognized and whose communication may not have been intended. Using this type of analysis may help the clinician to reconsider and reformulate elements of the analytic process that have previously been out of awareness, and may potentially enable a fuller comprehension of the analytic interchange.

The measures to be applied here have been developed in the context of the theory of the Referential Process (RP), as formulated within Multiple Code Theory (MCT). This process incorporates the functions of Arousal, Symbolizing and Reflecting/Reorganizing, as these play out in the analytic interchange. Computerized measures of two of these functions have been developed and validated; see Bucci (2021), Maskit (2021).

## The Use of Clinical Reports in Psychoanalysis and the Empirical Evaluation of Linguistic Measures as Added Value

Analysts write about their analytic work for many reasons, such as for supervision, for their own edification, and/or to communicate their ideas to their colleagues (for example, Bernstein 2008; Stein 1988). In essence, the act of writing is an attempt to put into words something that takes place in the analyst's mind, arising from the experience in the encounter with another person, the patient. Analysts may also write about patients in order to work out and clarify for themselves something that troubles them about their analytic work with a particular patient; their writing in this way reflects the psychoanalytic process (Alstein 2016). In essence, writing about a case may be a form of self-analysis and/or self-supervision (Bass 2016).

The function of writing case reports was discussed in a recent issue of the *Rivista di Psicoanalisi* (Rossi Monti 2017; Balsamo 2017; Civitarrese 2017). In addition to a discussion of the various uses of case reports in the history of psychoanalysis, two questions were raised: (1) Why do clinicians feel a need to write about the information they obtain from their analytic treatments? And, (2) how do they use clinical material to develop theoretical perspectives?

Clinical reports shared with colleagues usually cannot convey the complexities of the therapeutic sessions. Freud, himself, commented on this difficulty. He said:

It is well known that no means has been found of in any way introducing into the reproduction of an analysis the sense of conviction which results from the analysis itself. Exhaustive verbatim reports of the proceedings during the hours of analysis would certainly be of no help at all; and in any case the technique of the treatment makes it impossible to draw them up” (Freud 1918, p. 13).

Yet, psychoanalysts continue to write and /or to tell about their experiences. On one hand, there is theoretical and clinical utility to the field when analysts write and talk about their clinical work. On the other hand, there are many personal reasons for writing about clinical cases. For example, Balsamo (2017) notes that case reports should communicate to the reader the essence of the therapeutic work, even though the clinical material is conceptualized subjectively by the analyst. The post-session subjective selection of data is compiled in such a way that a coherent story emerges, dependent on the analyst’s theoretical and personal perspective.

### Comparison between Clinical Aspects and Linguistic Styles of the Analyst's Notes on a Single Case

A wealth of theoretical models and different paradigms has always characterized the field of clinical psychoanalysis. However, too often, there has been an antagonism between clinical and systematic empirical approaches rather than an integration of such methods (Eagle and Wolitsky 2011). The utilization of linguistic measures along with clinical evaluation is one example of an integration of clinical methods with a systematic empirical approach (Hoffman 2019).

Using Multiple Code Theory (MCT) as a framing perspective, several studies have shown that application of the linguistic measures to clinical notes enhances the understanding of the subjectively compiled notes (Bucci et al. 2012; Hoffman et al. 2013). In the latter publication, the authors suggest that an evaluation of the trajectory of two analytically relevant linguistic variables applied to notes could add to the understanding of the clinical analytic work. The two variables are Mean High WRAD (MHW), a measure of the degree to which language is connected to emotional experience, and Reflection (REF), the use of words referring to thought and logical functions. The authors compared a successful case and an unsuccessful case as evaluated by judges, who were blind to the treatment outcome. They found that changes in the relative position of the MHW and REF variables indicated **nodal points** in the treatment which then could be subject to an in-depth clinical evaluation.

These portions of the treatment would not necessarily have been noticed during a standard clinical review of the case notes. A nodal point which indicated a therapeutically helpful situation in the successful case was marked by an alternation of the position of the two measures. While MHW is generally higher than REF, there was a period in which REF became higher; then, at a nodal point in the interaction, following some time in which REF was greater, MHW again rose above REF. In contrast, a nodal point that indicated a problematic situation in the unsuccessful case was marked by a persistence of the REF staying higher than MHW. An in-depth clinical examination of the analytic material around these nodal points provided probable analytic reasons for the success of one treatment and the lack of success of the other. The analyst who described the analytic interaction with greater emotional immersion, in fact, had greater success. One wonders whether such linguistic analysis implemented contemporaneously, and available to the treating analyst, might have changed the course of the unsuccessful case.

The major goal of this paper is to carry out an application of the empirical/clinical approach outlined above, in which the treating analyst herself applies the linguistic measures to the notes and examines the results. We hypothesize that a clinical report can represent a Referential Process (RP) within the analyst, which can be evaluated by examining the linguistic measures. Verbal and non-verbal communications by the patient in the consulting room stimulate subsymbolic bodily and emotional experiences within the analyst that have not yet been formulated (the Arousal function). These lead to an internal narrative that may involve images and/or words (the Symbolizing/Narrative function), followed by processes of Reflection and Reorganization. The analyst’s theoretical and personal perspective determine the nature of his or her reflection.

This internal process within the analyst, which is then translated into a clinical report, is similar in its components to the ideal case report described by Bernstein (2008). He notes that such a report consists of “a repeating three-part structure… of an experiencing section of several paragraphs, in which the writer describes the experience-near analytic interaction over a relatively circumscribed time period; a reflecting section, in which the writer draws back for an overview of this material; and a transitional narrative section, in which the writer creates a bridge to a time later in the analysis” (page 433),

This study highlights how examination of the trajectory of linguistic variables helped identify narrative phases and reflecting/reorganizing phases and their relationship in the clinical notes. A review of these linguistic findings enabled the analyst to perceive elements in the analytic work, including pointing to nodal moments in the analytic process, which were previously outside of her awareness.

This study is a retrospective examination of the notes of analytic material, notes that were collected by the analyst for her personal use as part of standard procedure during clinical psychoanalysis. The research project presented here, applying linguistic measures on the notes, occurred some years after the treatment. A major goal of this retrospective examination is to investigate the potential effects of such a linguistic analysis carried out contemporaneously with a treatment. Would elements emerge of which the analyst is unaware? Would this help the progress of the treatment in subsequent sessions? Or, perhaps, would elements that are highlighted by the linguistic analysis appear irrelevant or even derail the internal exploration of the analyst and consequently of the patient? We address these questions following the presentation of the case material.

### The Individual Case from a Clinical Point of View

M. was a long-distance trucker who was thirty years old at the time of entering treatment. He came into treatment because he could no longer manage to work. He quit his previous job impulsively without knowing what his next step would be. He remained lying on his couch, overcome with paralyzing anxiety. His body was covered with a skin rash associated with psoriasis, requiring pharmacological treatment. In addition, there was frequent abuse of drugs. Often in the sessions, he stated "I don't feel I am alive, sometimes I think I am like an alien.” This overall profile, including the somatoform symptoms, was consistent with the DSM-5 diagnosis of Depersonalization/Derealization Disorder (DDD) (American Psychiatric Association 2013), which is characterized by feelings of unreality of the outside world along with a feeling of foreignness of one’s own body or parts of it.

Bouvet (1960) defines the depersonalization which occurs in DDD as that moment when a person observes one’s mental processes, actions, body and other people with the impression of dreaming. This state is described as the demolition of one’s own world of perceptions and of one’s spatial and temporal experiences. No consistency exists between subjective and objective time and there is no harmony between previous and present experiences. M.'s depersonalization was projected onto his past and, as a result, the memories of his experiences appeared unfamiliar, as if they belonged to someone else and his memories were fixed so that there was no room for conflictual aspects or for affection.

Drugs were an anesthetic used to calm his strong anguish. As a result. he was unable to think, increasing his apathetic state. The treatment was carried out at a frequency of, initially, three and then four times a week; the increased intensity was initiated by the patient. During the first two years of analytic work, there was a remission of the somatic symptoms, disappearance of the skin rash and discontinuation of the steroids. There was consequent physical improvement, weight loss, and discontinuation of the drug abuse. The following is a summary statement from the analyst’s notes^1^.

During these first two years the patient was unable to emotionally talk about himself, only about the travel in his work and the books he had read. He would simply recount his travels, describing the routes he would take, how he would get lost, the nature of the petrol stations, problems with the wheels of the truck, and how often he needed to refuel. These narratives were used to create a common fabric, a shared language. He stressed that he liked his job “because at night there’s no traffic, and above all there is nobody around.” What unfolded, clinically, was a complex picture where the patient had difficulty focusing on internal aspects of himself, with a total absence of emotional self-narratives from the past or in the present. Furthermore, a strong sense of being alienated from the analyst prevented him from attending many sessions.

The analyst’s attention to the patient’s singular focus on things and facts was reminiscent of Ogden’s (2007) recommendation. Ogden discusses the importance of accepting, as is, the communications from patients who are not able to daydream and to associate freely. He differentiates “waking-dreaming” from “talking-as-dreaming.” Waking-dreaming manifests itself in the analytic setting in the form of free association. Talking-as-dreaming:

… is a loosely structured form of conversation between patient and analyst that is often marked by primary process thinking and apparent non-sequiturs. Talking-as-dreaming superficially appears to be ‘unanalytical’ in that it may seem to consist ‘merely’ of talking about such topics as books, films, etymology, baseball, the taste of chocolate, the structure of light, and so on. (p.575).

In fact, as Ogden notes, talking-as-dreaming serves as a form of waking-dreaming in which patients have been able to begin to dream formerly un-dreamable experience. The talk about technicalities of his job (petrol stations, the wheels of his truck, refueling) and their role in the referential process may be seen in this light.

## Method of Study Utilizing Linguistic Referential Process Measurements

The analyst collected reports of each session for the first two years, for a total of 198 notes, as a personal work method. As in the study of Hoffman et al. (2013), measures related to referential activity (RA) and Reflection were used for this linguistic analysis of the psychoanalytic process. Starting with the Italian Weighted Referential Activity Dictionary (IWRAD; Mariani et al. 2013)^2^ and the Italian Reflection Dictionary (IREF; Mariani 2009).^3^,^4^, the DAAP software (Maskit 2021) constructed four measures for each note. These were the Mean IWRAD (IWRAD), the Mean High IWRAD (MHIW), Mean IREF (IREF), and the IWRAD_IREF covariation (IWRAD_IREF); these are described below. As this last measure is reliable only for longer texts, very short notes were eliminated from this study. There were 159 notes long enough for linguistic analysis; the mean length of these notes is 729 words (SD = 30). These linguistic measures enabled the implementation of a retrospective consideration of the clinical process.

As described in several papers in this issue, the Referential Activity measures provide indicators of the extent to which narratives are being told in an emotionally immersed way. The DAAP software (Maskit 2021) compares each word of each note with the IWRAD, thus assigning a weight to each word. The first measure, IWRAD is the average weight across each note. For each note, DAAP produces a smooth curve showing where these IWRAD weights are generally higher and where they are generally lower. The MHIW is a measure of how high this curve is when it is high; it can be viewed as a measure of the intensity with which narratives are told. The MIREF is an overall measure of the extent to which the speaker uses words connoting reflection; and the IWRAD_IREF covariation, is a measure of the extent to which a speaker is simultaneously involved in narrative and reflecting on it.

A negative IWRAD_IREF covariation is generally indicative of the presence of effective processing of an emotionally rich remembered narrative. A negative covariation between WRAD and REF in both English and Italian has been associated with therapeutic success (Bucci and Maskit 2007; Andrei et al. 2008; Mariani and De Coro 2013).

### Evaluation of M (Clinical + Linguistic Analysis)

In her summary evaluation of M, written before the project of applying the linguistic measures was planned, the therapist states:

At the beginning of our sessions, M.'s stories were descriptions of things, he was unable to talk about his feelings or his emotions but everything was displayed as if in an active theater, with a constant "doing" and "going." With him, I felt it was essential to constantly translate his movements into representations of how he worked internally; I gradually shared this language with him. He was unengaged in this decoding job, remaining passive; I found myself overcome by his stories of recurring actions. They lost meaning, and I was assailed with boredom. I was confused and as paralyzed as he was on the couch. I was beginning to glimpse change constantly in the sessions, not so much the increased time spent with his girlfriend or his greater capacity to carry out his commitments, certainly important elements. However, what I felt changing session after session was his mode of speaking and thinking, managing to orient a focus into his inner world and in parallel, an increased ability to allow fragments from the past to re-emerge. In particular, relations with other stories began to emerge, which we used to reflect on together. There was a remission of his symptoms of sleepiness and the engulfing apathy that prevented completing his commitments, along with a total suspension of medication for psoriasis, as he had no more symptoms, and abandonment of the abuse of drugs.

As discussed by Hoffman et al. (2013), a therapeutic process is most likely to be present if the linguistic analysis of the notes shows emotional involvement as relatively dominant and alternating with abstract language. Thus, if the linguistic analysis shows mainly a pattern of high IREF and low MHIW a therapeutic process is likely to be absent. In computerized Italian referential process research, as in this study, it was found that the presence of a negative covariation in the sessions between IWRAD_IREF was an important factor in the therapeutic process, whether the sessions were recorded (Mariani et al. 2013; Marian and De Coro 2013) or reported in clinical notes (Mariani and Negri 2015).

**Fig. 1:**
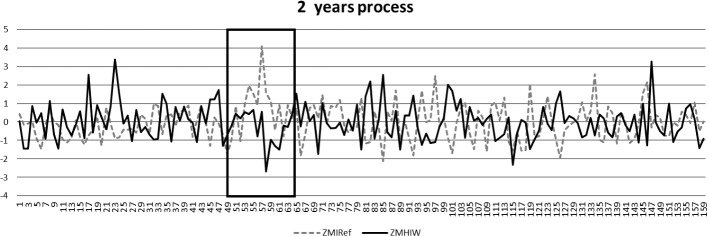
IREF pattern increased MHIW in the first year of treatment

**Fig. 2:**
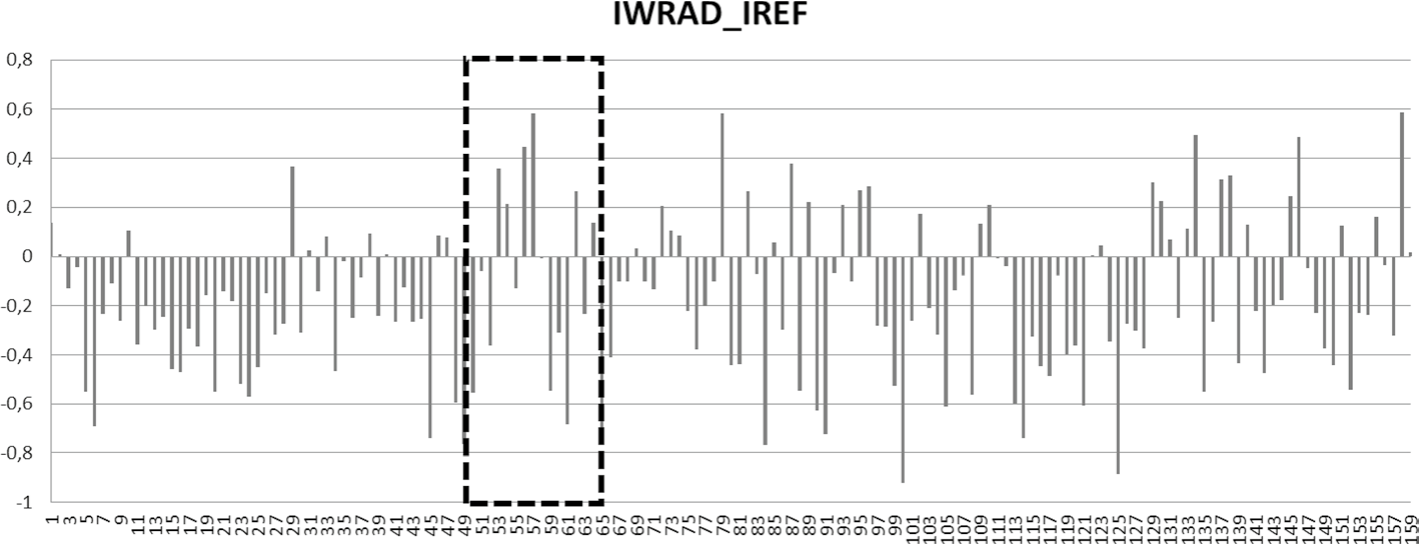
IWRAD_IREF covariation index applied to the notes in the two years of treatment

Observing the process over two years, as seen in terms of Z-scores of IREF and IWRAD, in a single graph (Fig. 1), one sees a continuous alternation of IWRAD and IREF. There are a few moments when this alternating pattern is absent for certain periods, after which the alternation is resumed. In Fig. 2, we observe the progress of the IWRAD_IREF covariation in the clinician’s notes. The covariation can vary between −1 to +1; for the two years shown, the mean of IWRAD_IREF was −0.18 (SD = 0.34); the covariation reached a minimum of−0.92 and a maximum of 0.59. One of the periods of alternating patterns, marked by the boxes in Fig 1, will be discussed in detail later in this paper

The linguistic indices on the whole, together with the large negative covariation, i.e., the presence of an alternation of high emotional immersion with logical, abstract language, confirm that in general there was an overall trend indicating the presence of a therapeutic/analytic process. This is confirmed by a negative correlation between IWRAD and IREF for the whole process of 159 notes (r =−267; p =.001). Below are examples of the different linguistic styles.

#### Example of High MHIW and Low IREF – Session Number 23

He remained in silence for a bit and then said, "Oh I dreamed of dying, or rather, as if I'd dreamed many times of dying ... the other day I was in the truck on the way home and as I passed over a viaduct I thought ... surely an accident here would make a nice flight, and just at that moment it was as if I had déjà vu ... like I had dreamed the same scene where I was falling into emptiness. And it is as if I had more repeated dreams like this...

#### Example of high IREF and Low MHIW – Session Number 57

He starts by telling me that he is having back pain, and that he is fine only in the truck. This comment takes me back to the beginning of the sessions. I think that privacy, maybe being alone in the house, causes his claustrophobic anxiety to re-emerge. After several minutes of silence, he tells me that he thought back to the dream and that a feeling of strangeness has remained. I keep hearing the dark symbolism of these dreams and the type of communication that he wants to have with me ... Then I ask him if between one session and another he happens to do some thinking about the sessions; he said: "not exactly thinking ... I'm not retaining anything; nothing remains for me, only the presence, the feeling, I don’t work on remembering things ... ideas only stay with me like this

Another example after one and a half years

#### High IREF and Low MHIW – Session Number 119

Now I live with the ghost ... I really feel double! The truth is that it is like a physical sensation, I feel torn in two moments, torn, I see myself in the third person. If I'm on the couch I feel a physical thing in my body, I get bored I mentally move away.” I am struck by the physical description and the description of split aspects. Then he says “but I still did not understand why all this is the cause that leads me to stay so where does this my inability to do things? I feel like I have to give him an answer. I tell him that I understand his desire as if to know the cause, point X, where things have taken another turn, and everything will be replaced. I deeply feel the frustration, the pain, the failure to understand and I feel close to the pain of not understanding.

#### High MHIW and Low IREF – Session number 126

He tells a dream: “I dreamed that I lit a fire throwing a cigarette in a forest, then I do not know if it was another dream or the same but I found myself having a clandestine relationship with a woman the feeling I felt was the same when I started attending X, we were lovers, in fact the woman had red hair like her. . . and he said in the dream the same things he told me at the time. how do we behave? “I think of the fire and the relationship of lovers and I tell him” in short, he would like to start a fire of passion and desire.” He laughs and says, “Yes, we cannot see each other… She’s always busy, then, if she’s around, I fall deeply asleep.” I think it is difficult for both to be able to have an intimacy of tenderness based on everyday life.

A review of the notes by the analyst led her to reflect on the two narrative styles. Why and when does each style occur? Can we conjecture that linguistic analysis of notes that exhibit high referential activity reflects analytic material which is well-understood by the analyst? For example, in the above example from session 126, after a year and a half of treatment, the analyst addressed the intimacy between analyst and patient. She writes “I think of the fire and the relationship of lovers and I tell him in short, he would like to start a fire of passion and desire.” He laughs and says, “Yes, we cannot see each other…” In the second type of notes, those with a high IREF, the report contains doubts, speculations, phrases that the patient says that leave different reflections open. For example, in the high IREF note, also from a year and a half, the analyst writes, “I feel like I have to give him an answer. I tell him that I understand his desire as if to know the cause, point X, where things have taken another turn, and everything will be replaced. I deeply feel the frustration, the pain, the failure to understand and I feel close to the pain of not understanding.”

This difference in the linguistic styles indicates a rhythm in the analysis, not only in the nature of the analyst’s understanding of the clinical material, but also as a reflection of the patient’s communications. For example, one of the main clinical characteristics of this patient was his difficulty in talking about himself. Sessions where he was open and expressive, as reflected in the analyst’s notes, appeared to alternate with memory gaps and absences. This pattern reflected his wish for closeness and his need to pull away. The alternation in the clinical report likely reflected the analyst’s experience of connection and distancing. This data is consistent with the idea that when the linguistic analysis of clinical notes shows an MHIW higher than REF, the speaker or writer is experiencing the moment with an immediacy and not reflecting on its meaning (Hoffman et al. 2012).

### Interruption and Recovery in the Analytic Process: The First Summer Break

The linguistic analysis points to a notable occurrence in the analytic process around the first summer separation, which occurred following Session 52 and the reunion in the fall (Session 53). The measures for the notes from this time period are marked by the boxes in Figs 1 and 2. From Session 53 to Session 64, the IREF is higher than the IWRAD; in some sessions markedly higher. Figure 2 shows that the IWRAD_IREF covariation in these sessions is positive (counter-indicating the presence of a Referential Process).

The linguistic pattern around the break can be considered to indicate a nodal moment, as described by Hoffman et al (2013). The persistence of higher IREF, that is, greater use of reflective words in comparison to referential activity in the process notes, would have been indicative of a potential problem in the analytic process. The identification of this nodal moment as reflected in the linguistic analysis would have enabled the analyst to uncover the nature of the analytic communication; she had not been fully aware of this shift in the process at the time of treatment. Here are excerpts from the notes concerning this period of the treatment.

The second session after the resumption after the summer (Session 54) is full of silences; his only communications relate to the description of what he did during the day. I feel very burdened; no dreams emerged. In fact, he tells me he had complicated and rich dreams but did not remember them. Then he begins the usual talk of how to reduce expenses. I start to feel a profound boredom [...] I feel very stuck in making interpretations; I feel that what comes to my mind is a bit predictable.

In session 55, he arrives on time and looks at me with a mischievous expression after having jumped a week of sessions. "Today I had to come because I had a dream that I had to tell you... I was in a place, I don't know quite where, with all men; then he says, I guess all naked men ... we were all naked and sat in chairs in a row ... kind of in a line-up and there were women for every man who came and gave oral sex ... and I was there and this girl just arrived and said to me...oh, no this isn’t going well! Everything has to be shaved or nothing!" now next to me is a guy who immediately gives me shaving foam and a razor to shave myself."

While I wait for his associations, I wonder why his dreams are often so eroticized. I feel that the dream contains many levels; on one hand, I think it has to do with us. Then I think about synchronicity, and I say, "maybe he feels that now that there is your space where he managed not only to have more intimacy but greater synchronicity ... though maybe he fears he won't be up to it." "maybe if... now instead it’s just her and me. I realize I am distracted in my thoughts and I try to pick up the thread of his talk. Towards the end of the session, he tells me about a fight with his girlfriend. He says, "but if we don’t share things why do we have to stay together ?!"

This excerpt from the process notes illustrates the confusion in the relationship between analyst and patient. It seemed as if the patient’s eroticization of the relationship was an attempt for him to seek contact with the analyst. Instead, the analyst was distracted and failed to address interpretively the impact of the summer interruption. Linguistic analysis of the notes indicates a lack of symbolization (low MHIW) and an increased use of intellectualized language (high IREF) by the analyst, including theoretical speculations and references to books, such as the following:

"This association strikes me because it came to my mind after the previous session and I really thought about the Dream Story book^5^. Despite this harmony of thought, I continue to find obscure the symbolism of these dreams and the kind of communication the patient wants to have with me."

Rereading the clinical notes after review of the linguistic measures allowed the analyst to understand more effectively the nature of the analytic process at this point. His feelings that the girlfriend did not share may have been a displacement from the transference, that the analyst was not there to ‘share things’ over the summer. Thus, the strong erotization of the field by the patient was likely a defensive structure on his part, provoked by the analyst’s response to moments where in the sessions, she distanced herself from him, reawakening her distance during the summer. Prior to the summer break there were also negative moments in the analytic sessions as well as missed sessions causing the analyst to worry about him. The patient’s expression of regressive, dependent, and passive desires towards the analyst provoked discomfort in the countertransference. As a way to deal with the countertransference issues, the analyst defended herself by becoming confused or finding theoretical anchors to try to recover a more therapeutic interaction with the patient. The analyst’s defensive confusion prevented her from thinking freely and fully. Reviewing the clinical material in the light of the linguistic measures enabled the analyst to put into words her doubts and her assumptions.

In fact, the writing of this case and the analyst’s reflection on the process can be considered as an example of the value of writing cases as described above: an emotional struggle within the analyst that is activated in the context of an analytic relationship but which struggles to be recognized. The case report stimulates a need to understand something that was not explicit in the analyst's internal process. This re-processing of the analytic interchange was amplified as a result of the linguistic analysis which pointed to a problematic area in the analytic material in this period.

Significantly, the analyst, during the treatment itself, was able to recover so that the notes from the following months (Sessions 61 to 70) indicated the presence of a Referential Process with lower IREF (See Figs. 1 and 2). For example, a month later, (Session 65, just before the IWRAD_IREF begins to become negative, Fig. 2) the notes state:

I'm going ahead with university practices; I managed to untangle the whole skein of physics and I'm trying to update my cv to present it to the conservatory, so I can enroll in the piano tuning course ... X is overjoyed because she saw the class schedule and we could attend together...

"I like this thing of going together ... This idea of being a piano tuner seems like a new movement towards a change of employment and I tell him that he might be trying to find a way to be closer. He tells me that that’s how it is ... I think what he's saying is important also in relation to missed sessions and loss of continuity that sometimes does not allow us to give meaning to things. I tell him and he nods and reiterates that this is how he feels when he stays still at home and can't manage to do anything.

This note shows recovery of a feeling along with movement, although still poorly understood by the patient. The distancing process expressed in the prior notes is no longer present in this material.

Many others have spoken of the therapeutic importance of vivid narratives. For example, Foa and Meadows (1997) have argued that the power of highly engaged narratives to express and activate emotion is widely recognized as a basis for exposure therapy. This has also been noted in psychological and neuroscience research (Damasio 2003; Ellsworth and Tong 2006; Harber and Pennebaker 1992). As Hoffman et al (2013) affirmed: “Such intense engagement may be discouraged in forms of supportive treatment in which the therapeutic goal is to modulate and downregulate the patient's affective intensity. Relatively lower MHW and higher REF indicate less emotional intensity and more emotional distance from the content by the speaker or writer” (p. 543). The effective operation of the referential process involves moments of emotional engagement in narratives followed by moments of reflection, in which emotions are examined, and reorganization and regulation may occur.

## Conclusion

This paper describes a multi-perspective process research design which incorporates the subjective experience of the analyst along with application of objective linguistic measures. Assessing the linguistic characteristics of the process notes is shown to enrich the traditional procedure of clinical reports.

Previous research on the referential process measures applied to the notes of clinical cases (Bucci et al. 2012; Hoffman et al. 2013) have identified specific linguistic configurations of productive or dysfunctional trends in the analytic process. In this paper, the measures applied by the analyst to her notes of a clinical case have confirmed this potential, highlighting elements of emotional patterns that came to life during the analysis, that were not noted at the time of treatment, and that supported retrospective reflection and understanding in the therapist. The notes at the time of the first summer break revealed a point of potential threat to the treatment that was not explicitly described at the time. In this case, the analyst was able to recover from this difficulty, as she describes. In other cases, such as one described by Hoffman et al. (2013), unrecognized difficulties around a similar nodal point led to an unsuccessful conclusion.

The use of notes provides a basis for integration of “on-line research” in the room with the patient, with “off-line research” involving empirical measures, without involving recording or assessment instruments in the treatment room. This methodology of integration between empirical and clinical research has considerable potential for analysts to support their search for meaning in the treatment room. In this way, they have a mirror on their referential processes, can increase their awareness of their way of working, and can elaborate emotional patterns arising in the exchange with the patient.

In this report we discuss a re-evaluation of clinical material based on retrospective application of linguistic measures to process notes of a completed case. The development of the new user-friendly DAAP software (Maskit 2018) enables the analyst to apply the measurements not only retrospectively, but in the treatment course, retracing and re-evaluating not only what *has happened*, but also what *is happening*. Such concurrent examination, like a variant of ongoing consultation, may open new directions for the therapist in reflecting on the progression of the treatment and guiding their work.

## Footnotes

1 Translated from the Italian by the analyst.

2 The IWRAD Dictionary contains 9,596 items, which cover about 95% of the text material used to construct and test it. Word examples included in the dictionary with their relative weights are: “che” (that) weight .00423; “non” (not) weight - .0357; “è” (is) weight .0162; “e” (and) weight .0542; “mi” (me) weight .0208; “a” (at) weight .0450; “di” (of) weight .0034; “perché” (because) weight -.01653.

3 The Italian Reflection dictionary is composed of words concerning how people think and communicate thoughts. This category includes words referring to cognitive or logical functions e.g., “supporre” (I assume), pensare (to think), pianificare (to plan) or entities e.g., motivo (reason), causa (cause), conseguenze (consequence); problems or failures of cognitive or logical functions e.g., confondere (to confuse); complex verbal communicative functions e.g., commentare (to comment), persuadere (to convince), discutere (to argue), offuscare (to obfuscate); features of mental functioning e.g., creative (creative), logico (logical). The total number of IREF words is 908, and it covers 3-4% of the spoken language.

4 A more recent measure of the Reflection function is the Weighted Reflecting/Reorganizing List (WRRL), developed using a modified version of the procedure used to develop the WRAD. It was not available at the time of this study.

5 The association was to Arthur Schnitzler book Dream Story or Rhapsody: A Dream Novel (German: Traumnovelle, 1926).
